# Glycine rich proteins of ticks: more than a cement component

**DOI:** 10.1017/S0031182024001410

**Published:** 2024-08

**Authors:** Renata Perotto de Souza, Mariana Vieira Dalla Valentina, Bruna Ferreira Leal, Sílvia Dias Oliveira, Carlos Alexandre Sanchez Ferreira

**Affiliations:** 1 Laboratório de Imunologia e Microbiologia, Escola de Ciências da Saúde e da Vida, Pontifícia Universidade Católica do Rio Grande do Sul (PUCRS), Porto Alegre, RS, Brazil; 2 Programa de Pós-graduação em Biologia Celular e Molecular, Escola de Ciências da Saúde e da Vida, Pontifícia Universidade Católica do Rio Grande do Sul (PUCRS), Porto Alegre, RS, Brazil; 3 Laboratório Central, Centro Estadual de Vigilância em Saúde da Secretaria de Saúde do Governo do Estado do Rio Grande do Sul (LACEN/SES-RS), Porto Alegre, RS, Brazil

**Keywords:** Tick salivary proteins, tick-host relationship, vaccine

## Abstract

Glycine-rich proteins (GRPs) are arbitrarily defined as those containing 20% or more glycine residues and constitute a superfamily divided into subfamilies based on their structure and/or function. GRPs have been identified in a diverse array of organisms and have been shown to possess a number of distinctive biological characteristics, including nucleic acid binding, adhesive glue-like properties, antimicrobial activity, involvement in the stress response and in the formation of cuticle components. In ticks, their expression has been described and studied mainly in the salivary glands, and their primary function is usually associated with cement formation and/or structure. Conversely, several GRPs are present in all tick developmental stages, and the expression of many GRP genes is modulated by physiological processes and immune challenges, such as feeding and pathogen infection. Considering that some tick GRPs appear to play essential roles in the tick life cycle, they have been evaluated as immune targets, with a focus on their potential application in vaccine development. This review highlights the roles that tick GRPs may perform beyond the formation and maintenance of the cement scaffold, including structural characterization, locations and functional relevance, hypothetical functions, and their potential use in anti-tick vaccine development.

## Graphical abstract

**Figure:**
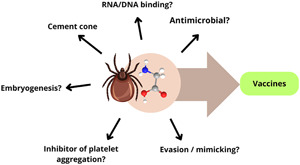

## Introduction

Glycine-rich proteins (GRPs) comprise many biomolecules, usually arbitrarily defined as proteins containing at least 20% glycine (Gly) in their composition. Typically, Gly amino acid residues are distributed in a glycine-rich domain that can be arranged randomly or in repeating patterns. Accordingly, GRPs are categorised into subtypes based on the pattern motifs they exhibit, which influence the protein structure and can allow for secondary structures such as alpha-helix, glycine loops or *β*-sheets. This affects the stability of proteins and may influence protein interactions and functions. Additionally, they facilitate the binding of other molecules, such as nucleotides (Bossemeyer, 1994; Sachetto-Martins *et al*., 2000; López-Llano *et al*., 2006; Dong *et al*., 2012). The functions of plant GRPs have been extensively studied, influencing plant development, physiology, and defence against abiotic and biotic stresses (Mangeon *et al*., 2010; Ma *et al*., 2021). In spiders and silkworms, GRPs are components of silk fibers, conferring flexibility with elastomericity and strength (Parkhe *et al*., 1997; Asakura *et al*., 2002; Dicko *et al*., 2008; Malay *et al*., 2016, 2017). In hemipteran insects, they exhibit antimicrobial and RNA binding activities (Futahashi *et al*., 2013; Meraj *et al*., 2024), while in spider mites and mosquitoes, GRPs have been implicated in feeding functions (Jariyapan *et al*., 2006; Sun *et al*., 2023). As a common structural component, GRPs are also found in insect cuticles (Zhong *et al*., 2006) and molluscan shells (Zhang and Zhang, 2006).

Ticks are hematophagous ectoparasites that are widely distributed across the globe and have evolved to thrive in a range of biotic and abiotic conditions (Ogden *et al*., 2021). They have a detrimental impact on both veterinary and human health, as they can transmit more pathogens than any other arthropod, including mosquitoes (Jongejan and Uilenberg, 2004). Moreover, the phenomenon of climate change has been suggested as the main cause for the spread of tick species to wider areas (Gilbert, 2021). Currently, tick control remains heavily reliant on the use of chemicals, an approach that has become increasingly unfavourable due to the emergence of resistant populations, environmental contamination, and rising costs (Obaid *et al*., 2022). Vaccines have been considered the most desirable alternative for tick control, and, although commercial vaccines were already occasionally successfully used, their development are still challenging (De La Fuente and Ghosh, 2024). In fact, anti-tick vaccine strategies must deal with caveats such as genetic diversity, immunodominant regions among protective antigens, development of long-lasting immunity and redundancy of functions among different molecules that can be recognized by the immune system (Leal and Ferreira, 2021; De La Fuente and Ghosh, 2024). Therefore, a cocktail of molecules may be more likely to achieve significant anti-tick protection (Ndawula Jr and Tabor, 2020), and some GRPs exhibit properties compatible with such desired multicomponent immunogens (Trimnell *et al*., 2002, 2005; Leal *et al*., 2018). The modulatory interplay between ticks and their hosts is likely to be the primary factor contributing to the parasite's success. A multitude of salivary proteins compose the biochemical dialogue between the two species, which undergoes changes over time, rendering tick saliva probably the most complex animal saliva (Ribeiro and Mans, 2020).

Ticks possess a wide variety of GRPs and their presence has been identified primarily in saliva and salivary glands (SGs), although their functions are not totally understood. Tick GRPs have been described as being involved in the formation of the cement, a hard glue structure secreted to attach to the host skin and hypothesized to help in the evasion of the host immune response, influencing pathogen transmission (Kemp *et al*., 1982; Havlíková *et al*., 2009; Hart *et al*., 2020). Ticks are ectoparasites that can be divided into longirostrate (long) and brevirostrate (short) according to the size of their mouthparts, which influences the amount of cement needed at the attachment site. Furthermore, the strategy employed by ticks to survive while feeding on a host can also vary according to the type of host, the number of hosts within the life cycle, or the duration of feeding on the host. In this context, the quantity and variability of GRPs appear to be influenced by the strategies of each tick species and developmental stage, in addition to the size of the tick mouthparts (Anderson *et al*., 2008; Maruyama *et al*., 2010). This review focuses on the structural aspects and dynamics of GRP production in ticks as well as discusses the possible roles these proteins may play in response to certain stimuli, such as feeding, infection and stress.

## GRP structural characteristics

**Figure 1.**
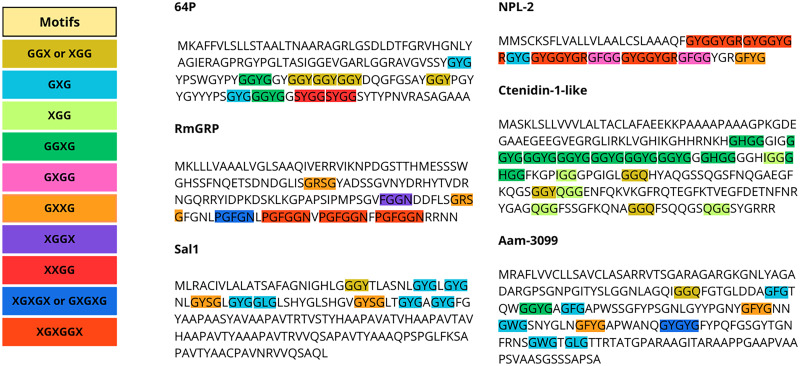
Gly-repeat patterns in tick GRP sequences. Each repeat is represented with a different color, and pattern variations within sequences are highlighted with the respective color. Sequences presented are: 64P of *Haemaphysalis longicornis* (AAM09648.1), RmGRP of *Rhipicephalus microplus* (AQX36208.1), Sal 1 of *Rhipicephalus annulatus* (AGR45924.1), NPL-2 of *Ixodes scapularis* (EEC15723.1), Ctenidin-1-like of *Ixodes scapularis* (XP_029830867.1), and Aam-3099 of *Amblyomma americanum* (JAG92486.1).

The content of Gly in the composition of GRPs varies between proteins, and Gly-rich motifs can be arranged in specific patterns or not. Glycine-rich regions can form loops, giving the region a flexible characteristic (Steinert *et al*., 1991; Högel *et al*., 2018). Gly residues themselves can also be displaced randomly or in patterns. Among ticks, the most common patterns described are tripeptides, such as GXG, GXX and GGX, but Gly-rich repeats can also be found in longer arrangements and in varying patterns, as shown in Fig. 1 (Bishop *et al*., 2002; Francischetti *et al*., 2009; Maruyama *et al*., 2010; Leal *et al*., 2018). For instance, the glycine-rich region of P29 of *Haemaphysalis longicornis* is present in the middle of the sequence and has been described as a collagen-like domain, as the GXX repeat that predominates resembles those present in vertebrate collagen proteins (Mulenga *et al*., 1999). The classification of GRPs within ticks has been primarily based on motif similarity, although it also been categorized by function, such as the cement GRPs, or by orthology, such as some *Rhipicephalus* GRPs, which are further categorized into distinct subclasses (GYG, expansion of large GYG, and superlarge GYG) (Francischetti *et al*., 2009; Ribeiro *et al*., 2011; Ribeiro and Mans, 2020).

As previously described, Gly residues are involved in the helical conformation of proteins, influencing the helix-helix packing, and, for instance, conferring greater stability to tertiary structures in transmembrane domains (Dong *et al*., 2012). Furthermore, the flexibility of proteins may be influenced by the position of Gly residues. Gly can lead to more flexible termini or rigid centres, which in turn alters the hydration and H-bonding of the helix (Högel *et al*., 2018).

At least three Metastriate ticks present GRPs containing GGY or GGY-related motifs, which, interestingly, present similarities with silk sequences of spiders (Mulenga *et al*., 2007; Maruyama *et al*., 2010). In dragline spider silks, these motifs are associated with mechanical characteristics, such as fibre strength, despite the glycine-rich region usually corresponds to a non-elastic domain (Brooks *et al*., 2008; Malay *et al*., 2016 and, 2017). In the insects *Bombyx mori* and *Antheraea pernyi,* the GGY sequence appears to be associated with cuticle hardening and plays a fundamental role in humidity-induced behaviour of silks (Suzuki *et al*., 2002; Futahashi *et al*., 2008; Wang *et al*., 2020).

The intrinsically disordered nature of many GRPs seems to facilitate liquid-liquid phase separation (LLPS) in the process of forming adhesive structures. In a salivary GRP from *Ixodes ricinus*, the N-terminal portion is more negatively charged, while the C-terminal portion has more aromatic residues regularly spaced by glycine-rich regions, which allows for extensive cation-π interactions and therefore the accumulation of particles around the GRP, leading to coacervation. Furthermore, it was demonstrated that the mature GRP (without the signal peptide), referred to as Tick-GRP77, undergoes LLPS and forms viscoelastic solid structures, as a transition to adhesive forms, what corroborates with a cement formation function (Ganar *et al*., 2023).

## Tissue and ontogenetic distribution in ticks

GRPs have been frequently identified in the tick cement, and their tissue localization has been studied in several tick species (Kemp *et al*., 1982). The components of cement are secreted in saliva, and the host species, feeding, and mouthparts morphology influence its composition (Tabor *et al*., 2017). Furthermore, GRPs presence is also modulated by biotic and abiotic factors (see Table 1). The following subsections present, compare, and discuss data on when and where these proteins are found and expressed in ticks, as well as the known conditions that may affect them.

**Table 1.** Distribution of glycine rich proteins in different tissues and developmental stages of ticks, and their response to feeding

Tick Species	Location	Developmental Stages	GRP levels and/or gene expression	Reference
Ixodidae				
*Amblyomma americanum*	Cement, salivary glands, midgut and saliva	Adult Female	Variable depending on feeding time	Bullard *et al*. (2016*a*), Radulović *et al*. (2014), Karim and Ribeiro (2015), Tirloni *et al*. (2017), Kim *et al*. (2020)
*Amblyomma cajennense*	Salivary glands	Adult Female	Up regulated during feeding	Maruyama *et al*. (2010), Garcia *et al*. (2014)
*Amblyomma maculatum*	Cement and salivary glands	Adult Female		Karim *et al*. (2011); Ribeiro *et al*. (2023)
*Amblyomma parvum*	Salivary glands	Adult Female	Up regulated during feeding	Garcia *et al*. (2014)
*Amblyomma sculptum*	Salivary gland and saliva	Adult Female	Up and downregulated during feeding	Esteves *et al*. (2017)
*Amblyomma triste*	Salivary glands	Nymphs and Adult Female	Up regulated during feeding	Garcia *et al*. (2014)
*Amblyomma tuberculatum*	Salivary glands	Adult Female		Karim *et al*. (2021)
*Amblyomma variegatum*	Cement and salivary glands	Adult Female		Díaz-Martín *et al*. (2013), Nene *et al*. (2002), Ribeiro *et al*. (2011)
*Dermacentor andersoni*	Salivary glands	Adult Female		Alarcon-Chaidez *et al*. (2007)
*Dermacentor variabilis*	Cement, salivary glands and midgut			Macaluso *et al*. (2006), Anderson *et al*. (2008)
*Haemaphysalis flava*	Cement and saliva	Adult Female	Present during feeding	Xu *et al*. (2016), Liu *et al*. (2023)
*Haemaphysalis longicornis*	Salivary glands, midgut, ovaries, synganglion, integument and carcasses	All Stages		Harnnoi *et al*. (2006)
*Haemaphysalis qinghaiensis*	Salivary glands and carcasses	All Stages		Jiang *et al*. (2014)
*Hyalomma dromedarii*	Salivary glands	Adult Male and Female	Higher levels in females	Bensaoud *et al*. (2018)
*Hyalomma marginatum*	Cement and salivary glands	Adult Male and Female		Francischetti *et al*. (2011)
*Ixodes holocyclus*	Salivary glands	Adult Female		Ong *et al*. (2016)
*Ixodes pacificus*	Salivary glands	Adult Female		Francischetti *et al*. (2009)
*Ixodes ricinus*	Cement and salivary glands	Nymphs and Adults		Perner *et al*. (2016)
*Ixodes scapularis*	Cement, salivary glands and saliva	Nymphs and adult female		Valenzuela *et al*. (2002), Ribeiro *et al*. (2006), Tirloni *et al*. (2017)
*Riphicephalus annulatus*	Salivary glands	Eggs and adult female		Shahein *et al*. (2013)
*Riphicephalus appendiculatus*	Cement and salivary glands	Adult Male and Female	Upregulated during feeding and at higher levels in males	Bishop *et al*. (2002), De Castro *et al*. (2016)
*Riphicephalus bursa*	Salivary glands	Female	Induced expression or upregulation of some GRP genes after host attachment or during feeding . Downregulation of two GRP genes after tick attachment.	Antunes *et al*. (2018)
*Riphicephalus microplus*	Cement, salivary glands, synganglion, gut, fat body, ovaries and saliva	All Stages	Variable depending on feeding time. Usually described as higher level in females, however has higher levels in males feed in resistent host.	Untalan *et al*. (2005), Lew-Tabor *et al.* (2010), Maruyama *et al.* (2010), Tirloni *et al.* (2014), Leal *et al*. (2018), Garcia *et al.* (2020)
*Riphicephalus pulchellus*	Salivary glands	Adult Male and Female	Higher levels in males	Tan *et al*. (2015)
*Riphicephalus sanguineus*	Salivary glands, synganglion and saliva	Adult Male and Female		Anatriello *et al*. (2010), Lees *et al*. (2010), Maruyama *et al*. (2010)
Argasidae				
*Antricola delacruzi*	Salivary Glands	Adult Female		Ribeiro *et al*. (2012)
*Ornithodoros coriaceus*	Salivary Glands	Adult Male and Female		Francischetti *et al*. (2009)
*Ornithodoros moubata*	Saliva	Adult Male and Female	Higher levels in females	Díaz-Martín *et al*. (2013)

### Ontogenetic distribution

The GRPs RmGRP and Rm39 of *Rhipicephalus microplus* and Hq15 of *Haemaphysalis qinghaiensis* were detected in all stages of development (Maruyama *et al*., 2010; Jiang *et al*., 2014; Leal *et al*., 2018). Also, a substantial number of transcripts encoding cuticle-like GRPs were identified in *R. microplus* nymphs and females during feeding. These transcripts showed considerable heterogeneity, including the presence of GGY family transcripts exclusively in nymphs (Garcia *et al*., 2020). Shahein *et al*. (2013) identified open reading frames (ORFs) for three putative salivary GRPs in *Rhipicephalus annulatus* (RaSal1, RaSal2 and RaSal3). RaSal1 and RaSal3 were present in eggs after 12 days of oviposition, while RaSal2 was weakly recognized after 6 days (Shahein *et al*., 2013). In *Haemaphysalis longicornis*, anti-sera against the GRP P29 reacted with larval and adult extracts, indicating its presence (or of immune-related proteins) in immature and mature ticks, while HLMLP (*H. longicornis* muscle LIM protein) was present in all developmental stages (Mulenga *et al*., 1999; Luo *et al*., 2020). Expressed sequence tags (ESTs) of GRPs have been identified in nymphs and adults of *Ixodes scapularis*, exhibiting considerable similarity to the *Ixodes pacificus* profile (Ribeiro *et al*., 2006). In *Amblyomma americanum*, five profiles of GRPs were detected in larvae and nymphs, with upregulation observed following ecdysis (Hollmann *et al*., 2018).

The profile of the GRP sialotranscriptome in ticks differs between males and females. This can be explained by the distinctive characteristics of attachment presented by each biological sex and presuming a higher necessity for variability and abundance of these proteins in females (Díaz-Martín *et al*., 2013; Tan *et al*., 2015). This assumption is corroborated by the findings that *Hyalomma dromedarii* (Bensaoud *et al*., 2018, 2019) and *Ornithodoros moubata* (Díaz-Martín *et al*., 2013) present a greater number of GRPs in females than males. In *R. microplus*, females exhibit a greater abundance of cuticle-like GRP transcripts, whereas two subtypes of GRPs were found almost exclusively in males (elastin-like and glue-like) (Garcia *et al*., 2020). On the other hand, this does not seem to be a universal fact. The transcriptome of *Rhipicephalus pulchellus* males shows almost threefold more transcripts of GRPs, while females exhibit greater variability in sex-exclusive GRPs (Tan *et al*., 2015). Additionally, *Rhipicephalus appendiculatus* males also presented twice the number of GRPs transcripts than females, suggesting that these proteins play roles beyond the cement formation, as males typically exhibit a shorter period of attachment to the same host point (de Castro *et al*., 2016).

### Distribution within ticks

GRPs may be present in many tissues and organs of ticks. GRPs were identified in the ovaries, fat body, synganglia, and midgut of *R. microplus* (Lew-Tabor *et al*., 2010; Leal *et al*., 2018) and *I. scapularis* (Lees *et al*., 2010). In *H. longicornis*, Hlim2 was present in all organs investigated, except for the midgut. In contrast, Hlim3 was identified in the SG, synganglia, and carcass (Harnnoi *et al*., 2006). Therefore, GRPs have a highly heterogeneous distribution, with some GRPs being present or uniquely associated with the SG (see Table 1) and the cement (Lees *et al*., 2010; Leal *et al*., 2018; Ribeiro *et al*., 2023).

The SG transcriptome of different tick species (*Dermacentor andersoni*, *R. appendiculatus*, *R. microplus, Amblyomma variegatum, Amblyomma tuberculatum* and *Antricola delacruzi*) and the proteome of *A. americanum* saliva reveal that GRPs transcripts and putative GRP proteins are abundantly present (Alarcon-Chaidez *et al*., 2007; Karim *et al*., 2011; Ribeiro *et al*., 2011, 2012; de Castro *et al*., 2016; Kim *et al*., 2020). The transcriptome of *Amblyomma maculatum* SG also encodes 12 types of GRPs (Ribeiro *et al*., 2023). However, *Ixodes holocyclus* presents less than 20% of their SGs transcripts for putative secreted proteins and therefore also shows a lower abundance of GRPs compared to other ticks (Ong *et al*., 2016).

### Influence of feeding

The feeding process induces ticks to alter the composition of proteins secreted in saliva (Radulović *et al*., 2014; Tirloni *et al*., 2017). In this sense, GRPs of *A. americanum* are more abundant in the SG of partially fed females than in unfed females (Jaworski *et al*., 1990). During the feeding period, nine GRP genes of adult *A. americanum* ticks showed differential expression that varied according to the time since the onset of feeding. Among them, the gene of the GRP AamerSigP-41539 was almost exclusively expressed in unfed and 24 h post-feeding ticks. However, the genes of Aam-36909 and Aam-41235 reached their maximal expression after 48 h of feeding, whereas Aam-41540 and Aam-3099 did so after 72 h and 120 h, respectively (Bullard *et al*., 2016*a*). Kim *et al*. (2020) detected a highly cumulative relative abundance of GRPs in the saliva of *A. americanum* during the feeding process. Most of the GRP genes (90%) were shown to be upregulated after 48 h of feeding, with a maximum peak at 72 h. In *R. appendiculatus*, GRP 64P gene transcripts were not detected in unfed ticks, whereas in fed females, the maximum expression was observed after 24 h, with a subsequent decrease in expression levels following detachment. In males, by contrast, the expression reached maximum levels seven days after the beginning of feeding (Havlíková *et al*., 2009).

In a proteome analysis of *Haemaphysalis flava*, the GRPs identified were shared between partially and fully engorged females (Liu *et al*., 2023). Conversely, 17 GRP transcripts were detected in the SG transcriptome of *Amblyomma sculptum*. Of these, nine were up-regulated and two down-regulated after 24 h of feeding, while only three GRPs were identified in the saliva proteome, with only one present at higher levels in fed ticks (Esteves *et al*., 2017). In the saliva of *R. microplus* females, the proteome of saliva showed a higher abundance of secreted GRPs in partially than in fully engorged females (Tirloni *et al*., 2014). However, Leal *et al*. (2018) identified higher transcript levels of RmGRP in the SG of fully engorged females. In unfed larvae of *R. microplus*, ORFs of different putative GRPs were also identified (Untalan *et al*., 2005). The presence of different levels and diversity of GRPs pre-, during and post-feeding may imply additional roles for these proteins beyond cement formation.

Bullard *et al*. (2019) demonstrated that the silencing of the GRP AamerSigP-41539 gene resulted in a 20-fold increase in the bacterial load during the feeding of *A. americanum*. Also, after 5 and 8 days of feeding, the bacterial load was two-fold higher when the GRPs AamerSigP-41539 and Aam-40766 were depleted, respectively, suggesting that, within the SG, GRPs are involved in maintaining microbiota homeostasis (Bullard *et al*., 2019).

The type of host also affects the expression of GRPs. *R. microplus* fed on susceptible bovine hosts presented a higher secretion of GRPs, including more variability in GRPs (Maruyama *et al*., 2010; Garcia *et al*., 2020). *A. americanum* showed a higher presence of GRPs in the saliva when fed on rabbits compared to humans or dogs (Tirloni *et al*., 2017). In *I. holocyclus*, GRPs contigs were detected at lower levels in ticks feeding on domestic animals than when feeding on bandicoots (wild marsupials) (Ong *et al*., 2016). Furthermore, artificial feeding has been shown to alter the levels of GRPs in the midgut of *I. ricinus* and the cement of *A. americanum* (Bullard *et al*., 2016*b*; Perner *et al*., 2016).

Maruyama *et al*. (2010) identified more transcripts of GRPs in *R. microplus* and *R. sanguineus* (brevirostrata) than in *A. cajennense* (longirostrata), and the monoxenic tick, *R. microplus*, presented more GRPs contigs than heteroxenous ticks (*R. sanguineus* and *A. cajennense*). Also, de Castro *et al*. (2016) compared GRP transcripts of *R. appendiculatus*, *R. punchellus* and *Amblyomma* spp., where *Rhipicephalus* spp. presented GRPs in greater abundance than *Amblyomma* spp. (de Castro *et al*., 2016). Therefore, biological aspects of ticks influence the saliva protein diversity, including GRPs. It was suggested that brevirostrata ticks, which possess smaller mouthparts than longirostrata, secrete a greater amount of cement in order to remain attached to the host, and ticks of a single host (monoxenic) must produce a more diverse repertoire of GRPs than ticks that change hosts during their life cycle (Maruyama *et al*., 2010).

### Microbial interactions

Ticks are well-known vectors for the transmission of animal and human diseases, and the presence of pathogens such as *Theileria* spp. and *Babesia* spp. changes the sialotranscriptome and sialoproteome of ticks (Paulino *et al*., 2021; Schäfer *et al*., 2022). In this context, the tick immune system has been shown to respond to the presence of pathogens, including salivary proteins produced by ticks, the expression of which can be stimulated by the pathogen itself and may aid in its transmission (Kurokawa *et al*., 2020).

With regard to GRPs, the infection of *Dermacentor variabilis* with *Rickettsia montanensis* resulted in a reduction in Oi814-GRP gene expression in the ovaries, midgut, and SG, with a 52% decrease compared to uninfected ticks (Macaluso *et al*., 2003). In contrast, GRP upregulation was detected in the ovaries of *Rickettsia*-infected *D. variabilis* (Macaluso *et al*., 2006). Infection by *Theileria parva* of *R. appendiculatus* resulted in a nearly twofold increase in the expression of three GRPs (Nene *et al*., 2004), and *R. bursa* infected with *Babesia ovis* showed a GRP associated with the cellular matrix that was also upregulated (Antunes *et al*., 2018). A flagelliform silk protein (100Silk) was found to be reduced in *R. microplus* infected with *Anaplasma marginale*, and, when the respective gene was silenced, the level of tick infection decreased (Zivkovic *et al*., 2010). Accordingly, *R. microplus* infected with *Babesia* presented a downregulation of one GRP in ovaries (Rachinsky *et al*., 2007). These data provide evidence that tick protein production is modulated by various components of their microbiome, including pathogens (Kurokawa *et al*., 2020). However, the overall interaction of GRPs with tick pathogens remains unclear. As possible immune proteins, GRPs would be expected to be upregulated as part of a tick response to infection, although GRP transcription could be inhibited by the pathogen. Some GRPs may also potentially be upregulated by the pathogen, which could be explained as facilitating transmission to a new host.

## Possible roles of GRPs in ticks

As described in the previous sections, tick GRPs may present multiple functions. In a similar manner, four classes of GRPs and more than 12 functions for GRPs in plants have already been described (Mangeon *et al*., 2010). Tick GRPs are often associated with cement formation, especially those expressed in the SG. However, in other tissues they seem to perform alternative and/or unknown functions (Maruyama *et al*., 2010). The differences described for GRP variability and abundance between adult males and females also suggest additional roles, as males are expected to attach and detach more times than females during feeding and reproduction. In fact, *R. appendiculatus* and *R. microplus* showed more total GRP transcripts in males, suggesting potential involvement in other physiological processes (de Castro *et al*., 2016; Bensaoud *et al*., 2019; Garcia *et al*., 2020).

The cement cone of *R. microplus* was previously described as presenting a high content of glycine (Kemp *et al*., 1982). Thereafter, GRPs in the cement were suggested to promote the hardening of the adhesion structure (Bullard *et al*., 2016*b*). As shown above, this is strongly corroborated by the property of certain GRPs to form a gel-solid structure after LLPS (Ganar *et al*., 2023).

Another important function that may be performed by tick GRPs is the modulation of the host immune response. The similarity of some GXX/GGX repeats in tick GRPs compared to the Gly-rich motifs of vertebrate GRPs indicates a probable function on evasion of host defence/haemostasis by mimicking host skin components (Mulenga *et al*., 1999; Bishop *et al*., 2002). This hypothesis is endorsed by the increased inflammation at tick attachment sites in GRP-silenced ticks, which also shows increased haemorrhaging at the tick bite site (Hollmann *et al*., 2018). Additionally, Gly can be recognized by receptors and when Gly binds to anion channel receptors, the chloride conductance increases, leading to chloride influx, which causes the hyperpolarization of platelets, thereby inhibiting platelet aggregation (Schemmer *et al*., 2013). Indeed, Ribeiro *et al*. (2006) identified one GRP as a probable inhibitor of platelet aggregation.

GRPs are also described to bind nucleic acids and RNA-binding GRPs are associated with the development of embryos in a range of organisms, as well as in the ecdysis of insects (Zhong *et al*., 2006; Ciuzan *et al*., 2015). In zebrafish, GRPs are also involved in spinal cord regeneration (Ma *et al*., 2021). In ticks, the presence of GRPs in eggs was reported in *H. qinghaiensis*, *H. longicornis*, and *R. microplus*. Furthermore, silencing GRP genes has been shown to reduce hatchability and weight of larvae (Jiang *et al*., 2014; Leal *et al*., 2018; Luo *et al*., 2020). These data indicate that GRPs are important players in tick development and that further studies can elucidate the specific roles that GRPs play in these processes.

In response to stress, insect GRPs in the cuticle protect against temperature changes, allowing them to adapt to new environmental conditions (Zhang *et al*., 2008). Response to environmental stress mediated by *A. americanum* GRPs has also been suggested, as temperature changes (cold and heat), oxidative stress, and injury positively or negatively modulate the expression of GRP genes. Interestingly, gene expression of the GRP Aam-40766 decreased in the SG but increased when the entire tick was analysed. On the other hand, the expression of the GRP Aam-36909 gene was downregulated in the whole tick when exposed to cold or injury stresses, but upregulated in the SG, which was suggested as a physiological adaptation (Bullard *et al*., 2019). In eggs of *H. longicornis*, one GRP is also upregulated in response to temperature stress (25°C) (Luo *et al*., 2020).

Antimicrobial activity is a known function of GRPs and glycine-rich antimicrobial peptides (GR-AMPs), including silk GRPs from spiders, scorpions, and insects. GR-AMPs are molecules of the innate immune response that act against a wide range of pathogens. In general, GR-AMP sequences contain a signal peptide, a pro-peptide and a glycine-rich region, which usually present domains with repeats containing Gly residues (Yi *et al*., 2014; Wang and Wang, 2016), similar to what can be found in tick GRPs. Attacins, ctenidins, gloverins, diptericins, serrulins, acanthoscurrins, hyastatin, and prolixins are some GR-AMPs that have already been described and characterized (Yi *et al*., 2014; Wang and Wang, 2016; Meraj *et al*., 2024). Most of assays employing GR-AMPs indicated activity against gram-negative bacteria, although effects against gram-positive bacteria, fungi, protozoa (Meraj *et al*., 2024) and virus are also reported (Yi *et al*., 2014; Wang and Wang, 2016). For example, serrulin from the scorpion *Tityus* s*errulatus* inhibits the growth of *E. coli*, *Pseudomonas aeruginosa* (gram-negative bacteria), *Micrococcus luteus* (gram-positive bacteria), *Aspergillus niger* (filamentous fungus) and *Candida albicans* (yeast) at low concentrations and its sequence presented similarity to acanthoscurrins (Acantho 1 and 2) from the spider *Acanthoscurria gomesiana*, which present a reported antimicrobial activity (Lorenzini *et al*., 2003; de Jesus Oliveira *et al*., 2019). Interestingly, serrulin is also similar to a secreted GRP from the tick *I. scapularis* (de Jesus Oliveira *et al*., 2019), suggesting a possible antimicrobial role for this tick GRP.

GRPs/Gly-rich motifs may also present different activities not directly responsible for cellular killing. One such example is the GRP hyastatin of *Hyas Araneus*, which presents three domains: an N-terminal region containing Gly residues, a Pro/Arg-rich region and a C-terminal region containing six cysteine residues. Native hyastatin exhibits antimicrobial activity against bacteria (*E*. *coli* and *Corynebacterium glutamicum*) and yeast (*S*. *cerevisiae* and *C*. *albicans*), whereas the recombinant N-terminal region does not present the same effect, although both proteins are able to bind chitin. Therefore, it is suggested that the Gly-rich region may be primarily involved in pathogen attachment rather than in cell disruption (Sperstad *et al*., 2009). Insect attacins, the most extensively studied GR-AMPs, were first identified in the giant silk moth *Hyalophora cecropia* and are probably the most promising antimicrobials among the GRPs (Yi *et al*., 2014; Wang and Wang, 2016). Attacins may increase outer membrane permeability, bind LPS, and inhibit the synthesis of specific proteins, such as the OMPs, without reaching the internal membrane or cytoplasm (Carlsson *et al*., 1998). In terms of mechanism of action, attacins can be compared to polymyxin. The polymyxin-resistant strain of *P. mirabilis* EH193 was found to be sensitive to an attacin at a concentration 100-fold lower than polymyxin (Carlsson *et al*., 1998; Yi *et al*., 2014). Additionally, a peptide with 50% identity to *Bactrocera dorsalis* attacin B showed antimicrobial activity against methicillin-resistant *Staphylococcus aureus* (MRSA) (Shin and Park, 2019). Although the antimicrobial activity of tick GRPs was not directly tested for tick GRPs, it has been previously suggested that members of the GGY GRP family may possess this activity (Ribeiro *et al*., 2006; Mulenga *et al*., 2007; Francischetti *et al*., 2009). If tick GRPs do have antimicrobial properties, then modulation of GRP levels by pathogens may represent another component of the arms race in the host-parasite-vector relationship.

## Vaccine potential

Tick infestations and tick-borne diseases are emerging global health concerns for both livestock and humans. Conventionally, acaricides are employed to control these ectoparasites, but resistant ticks and the worrisome effects of chemicals emphasizes the need for the development of alternative strategies, such as vaccines (Schetters *et al*., 2016). In this context, tick GRPs have already been identified as protective antigens, and the outcomes of immunization protocols can be seen summarized in Table 2 (Trimnell *et al*., 2005; Shahein *et al*., 2013; Antunes *et al*., 2018; Couto *et al*., 2021).

**Table 2.** Glycine rich proteins from ticks used in immunization protocols, the respective outcomes and organ/tissue distribution

Proteins	Site of expression/presence	Efficiency	Species	References
p29	Salivary glands	Reduction in 16.5% of engorgement weight in adult females, and 40 and 56% mortality of larvae and nymphs post-engorgement, respectively.	*H. longicornis*	Mulenga *et al*. (1999)
HL34	Saliva	Reduction in oviposition (7.9%) and increased mortality rates of 14.7% for nymphs and 29.1% for adults. The morphology of dead ticks was altered, smaller and reddish in color.	*H. longicornis*	Tsuda *et al*. (2001)
HLMLP	Integument, salivary glands, midgut and ovaries	A 20% increase in mortality rate and a 17.44% reduction in engorged weight were observed in adult females.	*H. longicornis*	Luo *et al*. (2020)
Hlim2	Predominantly in the salivary glands, but present in all organs except the midgut	Reduction of 10% on engorged body weight of adult females.	*H. longicornis*	Harnnoi *et al*. (2006)
Hlim3	Predominantly in the salivary glands, synganglion and carcass	Reduction of 66% of attachment rate 24 h post-infestation, and prolonged engorgement time.	*H. longicornis*	Harnnoi *et al*. (2006)
RH50	Salivary glands of fed ticks	Reduction of 25.3 and 13.5% on attachment rate of adults and nymphs, respectively. Increase of 30.5% in the mortality rate of nymphs.	*R. haemaphysaloides*	Zhou *et al*. (2006)
Rm39	Salivary glands	Reduction of tick's feeding time, number of adult females (52.5%) and mean engorgement weight (55.2%).	*R. microplus*	Maruyama *et al*. (2017)
64TRP	Salivary glands	The mortality rate of nymphs post-attachment increased 48% when immunized with 64TRP-6. The mortality of adult female ticks post-detachment increased 55.5, 70, and 60%, respectively, following immunization with 64TRP-2, 64TRP-3, and 64TRP-5.	*R. appendiculatus*	Trimnell *et al*. (2002)

P29, a GRP of *H. longicornis*, was used for immunization of rabbits, reducing the feeding time and increasing the mortality of larvae and nymphs (Mulenga *et al*., 1999). A recent study used HLMLP, a cysteine-glycine-rich protein, to immunize rabbits, affecting engorgement (Luo *et al*., 2020). Two other *H. longicornis* GRPs were used to immunize mice. Hlim2 reduced the weight of engorged females, while Hlim3 reduced tick attachment and duration of feeding time (Harnnoi *et al*., 2006).

Another potential antigen candidate is RH50 from *Rhipicephalus haemaphysaloides*. In ticks fed to RH50-immunized rabbits, the attachment of nymphs and adults decreased, and nymphal mortality increased (Zhou *et al*., 2006). Vaccination of Holstein calves with Rm39, a GRP of *R. microplus*, resulted in weak immune recognition, and significant anti-Rm39 IgG levels were only obtained at the end of the challenge. However, egg weight and hatch rate were reduced in vaccinated calves, while fed adult ticks were capable of full engorgement, albeit with a pale colour (Maruyama *et al*., 2017).

Mice immunized with the *R. appendiculatus* salivary protein *64TRP* reduced the transmission of tick-borne encephalitis virus (TBEV) when transmission occurred from infected mice to nymphs and infected ticks to mice. Also, secreted cement proteins demonstrated an anamnestic response after 64TRP vaccination (Labuda *et al*., 2006). In guinea pigs, the immunization with 64TRP reduced the attachment rate of *R. sanguineus*, and the vaccination with three different recombinant protein versions of truncated 64TRP fragments (64TRP2, 64TRP3 and 64TRP5) reduced the attachment of *R. appendiculatus*. *I. ricinus* fed in rabbits immunized with 64TRP2 reduced engorged weight and egg mass weight, and increased mortality, whereas immunization with 64TRP2/6 increased mortality of nymphs fed on guinea pigs. The feasibility of a broad-spectrum 64TRP-based vaccine against four ticks (*R. appendiculatus*, *I. ricinus*, *A. variegatum* and *R. microplus*) has been suggested based on the presence of cross-reactive conserved protective epitopes (Trimnell *et al*., 2002, 2005).

Other GRPs have been identified as potential vaccine candidates, but further studies are needed to assess their antigenic and immunogenic effects in the host and tick (de la Fuente and Contreras, 2022). Proteins present in tick saliva are interesting targets, as they must interact directly with the host immune system. Moreover, an increase in tick-borne disease transmission and tick territorial dispersal, both of which are influenced by climatic changes, highlights the potential health risks that ticks may pose to the animal food chain and public health (Madison-Antenucci *et al*., 2020; Tardy *et al*., 2022). This reinforces the necessity for the development of anti-tick vaccines, particularly those of broad-spectrum.

## Conclusions

GRPs are a very diverse group of proteins that have been reported to be important cement proteins in ticks, but the amount of data accumulated on them has shown that their roles must be much broader, especially when compared to what has been described in other organisms. This review focused on the features that may make these proteins a hallmark of the tick-host relationship. GRPs can be found with specific domains and repetitions of amino acid residues, often showing similarity to host proteins such as collagen, configuring probable modulators of the haemostatic and immune systems. The intrinsically disordered nature of some GRPs may be essential for LLPS, which seems to be mechanistically involved in the scaffolding of the tick adhesive structure with flexibility plus rigidity. The large variation in the presence of the different GRPs when evaluated ontogenetically, in different sexes, in the localisation of different tissues/organs of the tick, during feeding, and even when comparing genetically closely related species, suggests possible roles in additional physiological processes. The regulation of GRPs in response to injury, infection, oxidative stress and temperature changes also suggests an involvement in adaptation to different conditions or environments. Furthermore, it seems probable that tick GRPs are involved in nucleic acid binding and antimicrobial activities. In light of the aforementioned context, it seems unlikely that GRPs are involved in more than one type of physiological process in ticks. Indeed, it seems reasonable to suggest that many GRPs may be multifunctional proteins. It can therefore be concluded that GRPs are promising candidates for the composition of anti-tick vaccines. Although anti-GRP responses do not appear to increase the mortality of adult ticks, they significantly reduce the number of progeny. In order to improve the design of vaccination approaches and ultimately achieve more effective protective outcomes, it would be beneficial to gain a more comprehensive understanding of the roles and mechanisms of action of GRPs, as well as their dynamics within hosts and pathogens.

## Data Availability

Not applicable.
